# FASEB BioAdvances—Introductory editorial

**DOI:** 10.1096/fba.2019-00043

**Published:** 2019-06-13

**Authors:** Ralph A. Bradshaw

**Affiliations:** ^1^ University of California Irvine CA USA

**The Times, They are A‐Changin…**

Several years ago, the Federation of American Societies For Experimental Biology (FASEB) decided to found a new journal to further serve the broad spectrum of members of its constituent societies and the varied aspects of biological sciences they represent. FASEB BioAdvances was officially launched in January of this year under the leadership of Jasna Markovac, the Chair of the FASEB Publication and Communications Committee, and is now approaching the mid‐point of its first year of publication. It is a fully open access journal and is, of course, open to submissions from anyone, with or without a connection to the Federation. In addition to reporting new research findings, FASEB BioAdvances is interested in enhancing scientific discussion and will consider papers that address reproducibility (or the lack thereof) or provide a forum for reporting negative results and the subsequent discussion thereof. It will also publish reviews of timely subjects presented in a balanced way or perspectives/opinion pieces that make a case for a new idea or support an old one.

In a time when there is a clear proliferation of publishing opportunities, it is reasonable to ask “Why add to this growing mélange? Aren't there enough journals out there?” We believe the answers to those questions are that science is growing, the scientific community is expanding (thus creating additional need) and a significant number of these new publishing opportunities can be classed as “predatory”, that is, published for profit without regard for the accepted standards of the scientific community. The Federation, in its wisdom, believes that there is a need for the type of journal that FASEB BioAdvances represents.

With FASEB BioAdvances successfully established, Jasna is now passing the baton to me, and I will take over as Editor‐in‐Chief on June 1. Happily she will remain on board as Founding Editor and will continue to supply sound advice as the journal moves forward. This transition will also mark a period of expansion with new additions to the ranks of both the associate editors and the editorial board. In so doing we, the editorial team, hope to expand the scope of the science covered and make FASEB BioAdvances an even more attractive place for publishing original work and thoughtful opinion pieces. We in turn promise to provide timely and meaningful reviews and responsive management of the editorial process. FASEB passed its century mark just a few years ago and it represents one of the most important alliances in the biological sciences. Our challenge is to make this journal equal in stature to the organization that sponsors it.

